# From the Ground Up: Esophageal Atresia Types, Disease Severity Stratification and Survival Rates at a Single Institution

**DOI:** 10.3389/fsurg.2022.799052

**Published:** 2022-03-09

**Authors:** Devon Michael Evanovich, Jue Teresa Wang, Benjamin Zendejas, Russell William Jennings, Dusica Bajic

**Affiliations:** ^1^Department of Anesthesiology, Critical Care and Pain Medicine, Boston Children's Hospital, Boston, MA, United States; ^2^Tufts School of Medicine, Tufts University, Boston, MA, United States; ^3^Harvard Medical School, Harvard University, Boston, MA, United States; ^4^Department of Surgery, Boston Children's Hospital, Boston, MA, United States; ^5^Esophageal and Airway Treatment Center, Boston Children's Hospital, Boston, MA, United States

**Keywords:** ASA, EA, LGEA, mortality, term, term-born infant, PRAm, premature

## Abstract

Esophageal atresia (EA), although a rare congenital anomaly, represents one of the most common gastrointestinal birth defects. There is a gap in our knowledge regarding the impact of perioperative critical care in infants born with EA. This study addresses EA types, disease severity stratification, and mortality in a retrospective cohort at a single institution. Institutional Review Board approved our retrospective cross-sectional study of term-born (*n* = 53) and premature infants (28–37 weeks of gestation; *n* = 31) that underwent primary surgical repair of EA at a single institution from 2009–2020. Demographic and clinical data were obtained from the electronic medical record, Powerchart (Cerner, London, UK). Patients were categorized by (i) sex, (ii) gestational age at birth, (iii) types of EA (in relation to respiratory tract anomalies), (iv) co-occurring congenital anomalies, (v) severity of disease (viz. American Society of Anesthesiologists (ASA) and Pediatric Risk Assessment (PRAm) scores), (vi) type of surgical repair for EA (primary anastomosis vs. Foker process), and (vii) survival rate classification using Spitz and Waterston scores. Data were presented as numerical sums and percentages. The frequency of anatomical types of EA in our cohort parallels that of the literature: 9.5% (8/84) type A, 9.5% (8/84) type B, 80% (67/84) type C, and 1% (1/84) type D. *Long-gap* EA accounts for 88% (7/8) type A, 75% (6/8) type B, and 13% (9/67) type C in the cohort studied. Our novel results show a nearly equal distribution of sex per each EA type, and gestational age (term-born vs. premature) by anatomical EA type. PRAm scoring showed a wider range of disease severity (3–9) than ASA scores (III and IV). The survival rate in our EA cohort dramatically increased in comparison to the literature in previous decades. This retrospective analysis at a single institution shows incidence of EA per sex and gestational status for anatomical types (EA type A-D) and by surgical approach (primary anastomosis vs. Foker process for *short-gap* vs. *long-gap* EA, respectively). Despite its wider range, PRAm score was not more useful in predicting disease severity in comparison to ASA score. Increased survival rates over the last decade suggest a potential need to assess unique operative and perioperative risks in this unique population of patients. Presented findings also represent a foundation for future clinical studies of outcomes in infants born with EA.

## Introduction

**Figure 1:**
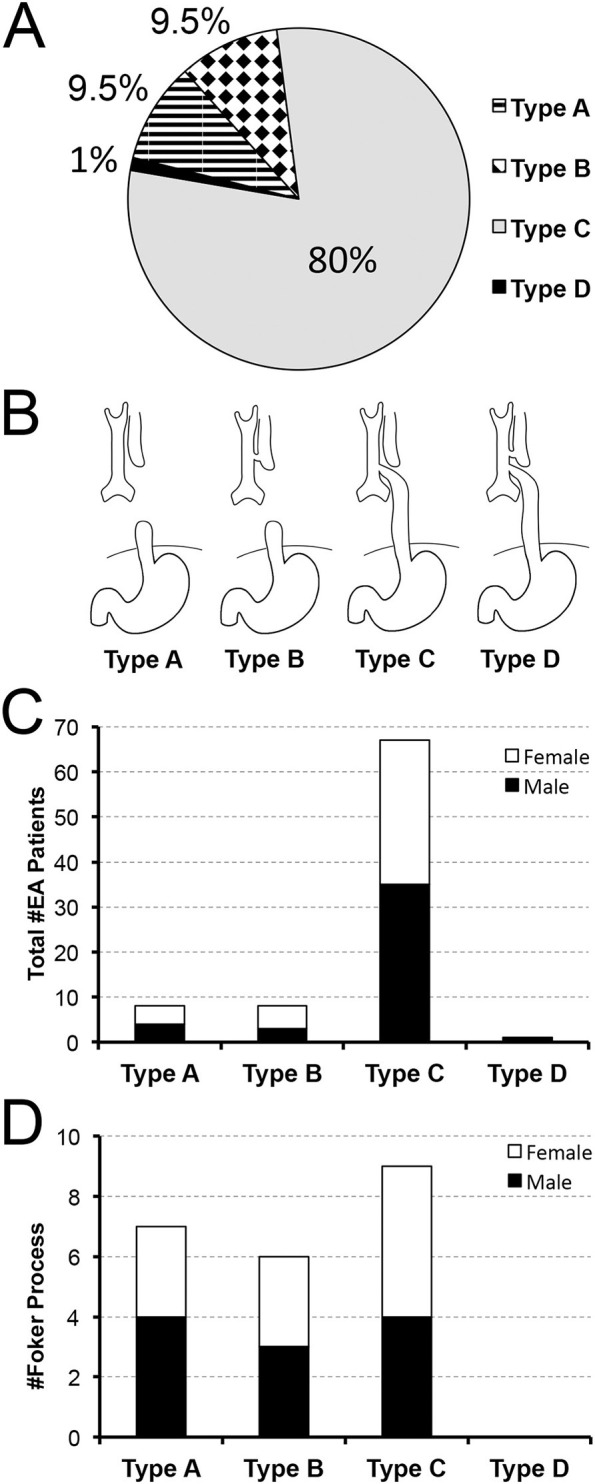
Retrospective Analysis of Incidence of Primary Esophageal Atresia Repair at a Single Institution. Data was retrospectively collected from 2009–2020 (*n* = 84). Pie chart in **(A)** summarizes the percent (%) incidence of esophageal atresia (EA) by anatomical classification that is schematically illustrated in **(B)**. According to our retrospective data analysis, anatomical *type C* of EA is the most frequently encountered. Graph in **(C)** also illustrates nearly equal sex distribution per anatomical type with females found in 50% in *type A*, 62.5% in *type B*, and 48% in *type C*. Graph in **(D)** summarizes the incidence of *long-gap* EA patients (*n* = 22) that underwent Foker process (5–8). Incidence of *long-gap* EA follows the trend per anatomical type and sex, similar to the cohort as a whole (see Results for more detail).

Esophageal atresia (EA), although a rare congenital anomaly with a stable world-wide prevalence (1) represents one of the most common gastrointestinal birth defects with reported incidence of 1 in 3,000 to 1 in 4,500 live births (2). If esophageal lumen interruption is left unrepaired, infants are prone to inadequate nutrition and growth, as well as infections such as pneumonia (3). EA is classified into 4 types (type A, B, C, and D) based on the anatomical description in relation to the airway structures (4) (Figure 1), that does not take into account the complexity of underlying disease.

In addition to associated malformation, the EA gap length dictates the complexity of perioperative care. If the gap of the esophagus is too large to be repaired by direct anastomosis (>3 cm or >2 vertebral bodies in length), EA is defined as *long-gap* EA, which is more commonly found in anatomical types A and B of EA (9). At our institution, the latter cases undergo a unique type of EA repair, the Foker process (5–8). Compared to *short-gap* esophageal atresia, *long-gap* EA is also more likely to be an isolated defect and associated with trisomy 21 (9). In contrast, *short-gap* EA is more commonly found with VACTERL anomalies (vertebral, anorectal, cardiac, tracheo-esophageal fistula and/or esophageal atresia, renal, and limb defects/ malformations) relative to *long-gap* EA (9). Last but not least, CHARGE syndrome (coloboma, heart defects, choanal atresia, growth retardation, genital abnormalities, and ear abnormalities) (10) can further complicate the care of infants born with EA.

The complexity of underlying disease with and without other congenital anomalies in the case of EA is undeniable. Although *short-gap* EA is repaired with direct anastomosis and requires shorter pain management, infants are vulnerable to post-operative feeding challenges (11) in addition to the impact of associated anomalies. In cases of *long-gap* EA, the revolutionary Foker process (5–8) encourages the natural growth and lengthening of infant's existing esophageal pouches, but it requires at least two separate thoracotomies/thoracoscopies with a subsequent prolonged postoperative intubation (12, 13) associated with development of physical dependence to drugs of sedation (13–15). The primary goal of this study addresses the severity of EA disease as a blueprint for a subsequent myriad of caregiving conditions that might impose challenges to developing infants born with EA. Specifically, we conducted a retrospective analysis at a single institution to analyze incidence of EA by severity of disease using American Society of Anesthesiologists (ASA) (16) and Pediatric Risk Assessment (PRAm) (17–19) scores in the context of (i) sex, (ii) gestational age (term-born and premature), (iii) anatomical classification of EA types, and (iv) the type of surgical repair (viz. direct anastomosis vs. Foker process with prolonged sedation). Our secondary clinical end-point measures looked into mortality risk in the context of other co-morbidities according to Spitz et al. (20) and Waterson et al. (21) classifications that take into account co-existing pneumonia and cardiac disease, respectively. This study was, in part, previously published as thesis (22).

## Methods

### Study Design and Subjects Equations

Institutional Review Board at Boston Children's Hospital approved this retrospective cross-sectional research study (IRB-P000007855) of infants born with esophageal atresia (EA) that underwent primary surgical repair at a single institution. The study conformed to the standards set by the Declaration of Helsinki and Good Clinical Practice guidelines. The patient information was obtained from a prospectively maintained clinical database, *The Esophageal and Airway Treatment Center* REDCap database, established in 2009. Considering our institution is not a birthing center, all infants cared for at our institution are considered outborn. Eligibility criteria included: (1) term-born (defined as birth between 37 and 42 weeks of gestation) and premature infants (28–37 weeks of gestation) born with EA of any type, and (2) patients that received their primary surgical repair at Boston Children's Hospital. Cohort patients underwent surgery in the first month of life with exception of two patients that were born outside of the State and underwent primary surgical repair at our institution at 2 and 3 months of age. Exclusion criteria included: (1) extreme prematurity (<28 weeks of gestation), and (2) any surgical repair at other institutions (including but not limited to EA repair). Our retrospective study included a total of 84 patients (*n* = 53 term-born; *n* = 31 premature) over the period of 11 years (2009–2020).

### Chart Review

Electronic medical record, Powerchart (Cerner, London, UK) was used to collect demographic data (viz. date of birth; gestational age at birth (weeks); birth weight (kg)) and clinical data. The latter included several end-point measures.

#### Esophageal Atresia Types

In addition to classification of EA into 4 anatomical types (type A, B, C, and D) based on the anatomical description in relation to the airway structures (4) – in particular to co-existence with tracheo-esophageal fistula (TEF), we also classified EA cases based on the length of EA gap into: *short-gap* (that was repaired by primary anastomosis) and *long-gap* that underwent repair by Foker process (5–8). Some of the patients with *long-gap* EA were managed with our newer minimally invasive Foker process which entails an internal adjustable traction system that is adjusted every 5–7 days via a thoracoscopy. As such, it leads to less postoperative muscle paralysis and sedation in comparison to the external traction Foker process via thoracotomy (8, 23, 24). For the purpose of this study, we identify *short-gap* EA with direct anastomosis repair, and *long-gap* EA with the Foker process repair - as these are the two main surgical approaches at our Institution.

#### Medical/Surgical Comorbidities

As EA often presents with other congenital anomalies and/or co-morbidities, we collected clinical data regarding any other genetic or chromosomal anomalies (e.g., Trisomy 18, 21 etc.), and any other associated congenital anomalies (e.g., vertebral, cardiac, anal anomalies etc.), some of which are a part of the complex congenital syndromes associated with EA such as VACTERL (25) or CHARGE syndrome (26). Co-existing cardiac anomalies were classified as minor (not requiring surgical intervention such as patent foramen ovale, patent ductus arteriosus, atrial septal defect, ventricular septal defect, dextrocardia), or complex (requiring surgical correction such as large ventricular septal defect, large atrial septal defect, coarctation of the aorta, large patent ductus arteriosus, and Tetralogy of Fallow). We quantified the incidence of other associated co-morbidities such as: (i) pneumonia treatment, and (ii) cardiac surgeries, which both served as a basis for mortality risk evaluation (see below). Due to the retrospective study design, characterization of associated co-morbidities was obtained from the medical records as part of the clinical diagnostics and treatment.

#### Disease Severity

Complexity of clinical status in the context of other comorbidities was assessed using two scoring systems: American Society of Anesthesiologists (ASA) (16) and Pediatric Risk Assessment (PRAm) (17–19) scores at the time of EA repair surgery. Assigning an ASA Physical Status classification level is a clinical decision based on several factors (16) and represents the most commonly used assessment of system level disease severity by anesthesiologists (**Figure 3A**–*Table*). ASA scores are based on several factors and range from ASA I (normal healthy patient) to ASA VI (a declared brain dead patient) (16). In contrast, PRAm scoring is a relatively novel measure introduced in 2017 (17–19) that involves 5 scoring points: urgency of surgical procedure (+1), presence of at least one comorbidity (+2), presence of at least one indication of critical illness (+3), age <12 months at surgery (+3), and co-existing malignancy (+4) for a range of scores from 0 to 13. PRAm scoring has been designed as a less subjective assessment of disease severity for the use specifically in pediatric populations (scores 0 – 13; **Figure 3B**–*Table*).

#### Mortality Risk Assessment

Considering our retrospective data collection spans period of last 11 years (2009-2020), it was used for comparison to previously published survival rates in infants born with EA as per two different scoring systems: (i) Waterston et al. (21), and (ii) Spitz et al. (20). As originally described by Waterston et al. (21), this scoring system takes into account weight of the patient, co-existence of other congenital anomalies, and pneumonia. Scoring is described as low risk (**group A:** birth weight >2.5 kg with no or co-existing congenital anomaly or pneumonia), moderate risk (**group B:** birth weight 1.8–2.5 kg with co-existing mild pneumonia and mild congenital anomaly), or high risk (**group C:** birth weight 1.8–2.5 kg with co-existing severe pneumonia and severe congenital anomaly). To improve clarity and transparency with scoring definitions, we modified the original scoring by Waterson et al. (21) for moderate and high risk groups. In this report, the moderate mortality risk group (group B) included infants with co-existing *moderate* pneumonia (defined as receiving antibiotics), and/or a *moderate* congenital anomaly (viz. limb anomalies, cleft lip or palate, atrial-septal defect or small patent ductus arteriosus). High mortality risk (group C) in this study referred to infants with *severe* pneumonia (defined as requiring mechanical ventilation) and/or a *severe* congenital anomaly (viz. defined as making survival difficult or impossible without surgical repair). The second scoring system, described by Spitz et al. (20), takes into account weight and co-existence of a severe cardiac congenital anomaly. The risk is described as low **(I)**, moderate **(II)**, and high **(III)** risk of mortality with major cardiac anomalies defined as one that required medical or surgical treatment.

### Statistical Analysis

Data was presented as numerical sums and percentages for (i) sex and anatomical classification of EA, (ii) gestational age at birth, (iii) distribution of *long-gap* EA patients for sex and gestational age, (iv) distribution of congenital anomalies, (v) disease severity scores, and (vi) survival rate. PRAm scores were also presented as numerical sums and as boxplot distributions indicating median scores, first and third quartile ranges, and absolute values for minimum and maximum values.

## Results

The retrospective chart review included infants that underwent EA repair at a single institution over a period of 11 years (2009–2020; *n* = 84): 53 term-born, and 31 premature (born between 28 and 37 weeks of gestation).

### Demographic Information and Incidence of Esophageal Atresia Types

#### Anatomical Types

Figures 1A,B illustrate incidence of EA patients according to the anatomical types of EA (4): type A (isolated EA; 8/84, 9.5%), type B (TEF at the upper esophageal pouch; 8/84; 9.5%), type C (the most common type of EA with TEF at the lower esophageal pouch; 67/84; 80%), type D (the rarest type of EA with TEF at each esophageal pouch; 1/84; 1%). Our novel data implicate equal distribution of sex for infants born with EA (Figure 1C) with 49% (41/84) female and 51% (43/84) male patients of nearly equal distribution per anatomical types of EA: 50% (4/8) female with type A, 62.5% (5/8) female with type B, and 48% (32/67) female with type C.

#### Esophageal Gap: Short-Gap vs. Long-Gap

We also distinguished between *short-gap* and *long-gap* EA (see Method's section). The latter is equated to Foker process repair (5–8), that represented 26% of the cohort (22/84). Unlike the cohort as a whole (Figure 1C), the incidence of *long-gap* EA cases showed nearly equal distribution by anatomical types (Figure 1D): type C (9/22; 41%), type A (7/22; 32%), and type B (6/22; 27%). However, *long-gap* EA accounted for 88% (7/8) of patients in type A, 75% (6/8) in type B, and 13% (9/67) in type C (not graphically shown). Importantly, infants born with *long-gap* EA showed exactly equal distribution of sex (50%; 11/22 female) with nearly equal distribution per anatomical types of EA (Figure 1D): 42% (3/7) female with type A, 50% (3/6) female with type B, and 56% (5/9 female) with type C.

#### Gestational Age

**Figure 2:**
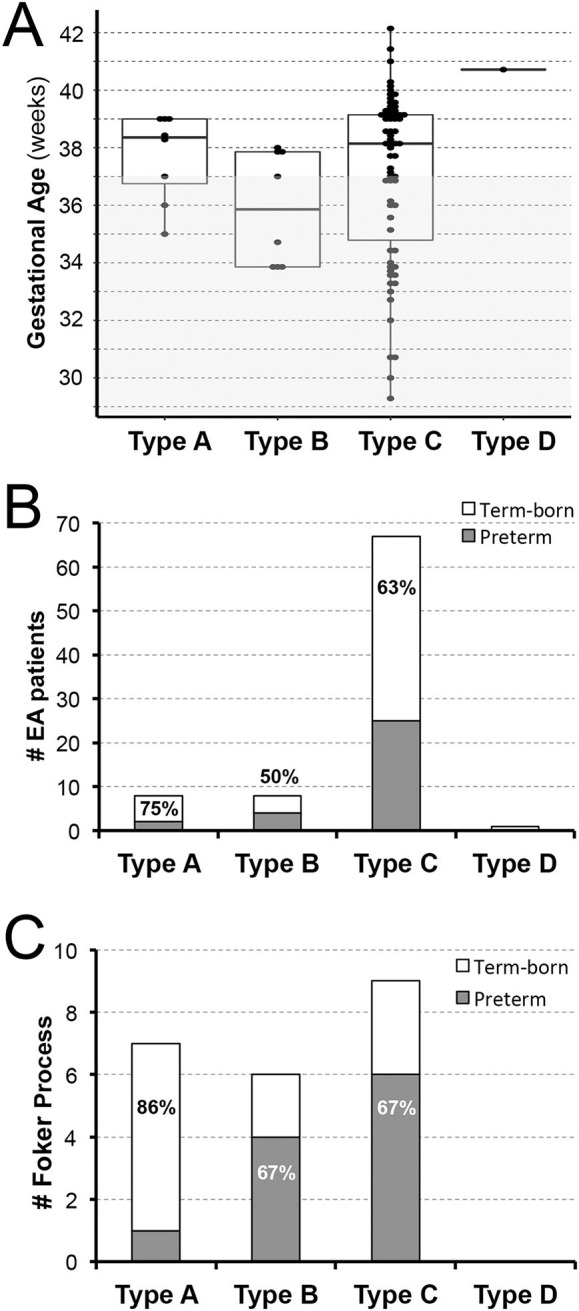
Retrospective Analysis of Esophageal Atresia Classification by Gestational Age at Birth. Retrospective data of infants born with esophageal atresia (EA) was collected from 2009–2020 (*n* = 84) and included infants born ≥28 weeks of gestation that were classified as term-born (37–42 weeks gestation) or premature (28–37 weeks of gestation). **(A)** Illustrates individual distribution of gestational age at birth per EA type (dots), while gray area schematically marks prematurity (<37 weeks of gestation). **(B)** Summarizes percent (%) incidence of EA per anatomical type and gestational age groups with either equal (type B) or predominant incidence of term-born patients (type A and C). In this cohort of infants with primary surgical repair of EA at our institution, we report only one term-born infant with *type D* EA. For illustration of anatomical EA types, please see Figure 1B. **(C)** Summarizes incidence of infants that underwent Foker process (5–8) for *long-gap* EA repair. We report equal incidence of term-born and preterm patients. However, term-born patients with *long-gap* EA were predominantly noted in *type A* (6/7; 86%) while premature infants with long-gap EA represented majority in *type B* (4/6; 67%) and *type C* (6/9; 67%) anatomical EA type.

Taking into account exclusion of extreme prematurity, this retrospective cohort shows slightly higher frequency of term-born (53/84, 63%) than premature patients (31/84; 37%) with similar trend per anatomical classification of EA types: 75% (6/8) term-born with type A, 50% (4/8) term-born with type B, and 63% (42/67) term-born with type C (Figures 2A,B). There was only one term-born patient with type D EA. Similarly, infants that underwent Foker process for *long-gap* EA repair (Figure 2C) had a similar frequency of term-born and premature patients (11/22; 50%). However, term-born patients with *long-gap* EA were predominantly noted in type A (6/7; 86%) while premature infants with *long-gap* EA represented majority in type B (4/6; 67%) and type C (6/9; 67%) as illustrated in Figure 2C.

### Incidence of Co-existing Congenital Anomalies

#### Syndromes Associated With Esophageal Atresia

Table 1 summarizes the incidence of other co-existing anomalies with EA. About 42% (35/84) of the cohort patients had complex EA disease as part of a syndrome or known chromosomal abnormality (Table 1A). Of those, the most frequent was VACTERL syndrome (31/35, 89%), although we also report cases of CHARGE syndrome (2/35, 6%), trisomy 21 (Down's syndrome; 1/35) and trisomy 18 (Edwards syndrome; 1/35). We also report a similar incidence of VACTERL syndrome in those that underwent primary repair (viz. *short-gap* EA; 24/62; 39%) compared to infants that underwent the Foker process for the repair of *long-gap* EA (7/22; 32%).

**Table 1:** Incidence of esophageal atresia in the context of other congenital anomalies.

	**Number**	**Percentage (%)**
**A. EA as part of complex congenital syndrome (cohort** ***n*** **=** **84)**		
VACTERL	31	37%
CHARGE	2	2%
Other	2	2%
None	49	58%
**B. EA with other co-anomalies apart from syndrome (*****n*** **=** **49)**		
None	6	12%
Isolated anomaly	10	20%
2 anomalies	17	35%
More than 2 anomalies	16	33%
**C. Distribution of co-anomalies apart from syndrome (*****n*** **=** **49)**		
Anorectal	0	0%
Vertebral	10	20%
Cardiac	38	78%
Laryngeal cleft	9	18%
Tracheo(broncho)malacia	23	47%
Limb	2	4%
Renal or Kidney	13	27%

***Incidence of esophageal atresia with or without other congenital anomalies. (A)** shows that 42% (35/84) of EA was a part of complex syndrome: VACTERL syndrome (31/35, 89%); CHARGE syndrome (2/35, 6%); Trisomy 21 (Down's syndrome; 1/35); and Trisomy 18 (Edwards syndrome; 1/35). As illustrated in **(B)**, of those esophageal atresia (EA) infants with no complex congenital diagnosis (49/84; 58%), a majority had either 2 (17/49; 35%) or > 2 (16/49; 33%) co-occurring congenital anomalies not associated with the syndrome. Incidence of specific co-existing anomalies is listed in **(C)**. Interestingly, a majority of patients had a cardiac anomaly (38/49; 78%) and no patients had a documented anorectal anomaly occurring outside of a syndrome. Acronyms: CHARGE, Coloboma, Heart defects, choanal Atresia, growth Retardation, Genital abnormalities, and Ear abnormalities; VACTERL, Vertebral, Anorectal, Cardiac, Tracheo-esophageal fistula and/ or Esophageal atresia, Renal, and Limb defects/ malformations*.

#### Esophageal Atresia in the Absence of Syndrome

For infants born with EA without associated syndrome (49/84; 58%), the majority had either 2 (17/49; 35%) or more than 2 (16/49; 33%) co-occurring congenital anomalies. Only a minority of patients had no co-existing congenital anomalies (6/49, 12%; Table 1B). Interestingly, a majority of patients – apart from syndromic patients (49/84; 58%) - had a cardiac anomaly (38/49; 78%) and no patients had a documented anorectal anomaly occurring outside of a syndrome (Table 1C).

#### Cardiac Co-anomalies

Of all the infants born with EA that had co-existing cardiac anomalies (72/84; 86%), only 18% (15/84) had congenital heart disease severe enough to require surgical repair (Table 2A). Of those that underwent cardiac surgery, 93% (14/15) had type C EA, and only one patient had type B EA. We report a similar pattern of co-existing congenital cardiac anomalies in infants with *long-gap* EA that underwent the Foker process repair (Table 2B): 86% (19/22) had co-existing cardiac anomalies, but only 14% (3/22) required cardiac surgery and were diagnosed with either type C EA (2/3) or type B EA (1/3). Table 2 summarizes severity of cardiac findings in this cohort.

**Table 2:** Incidence of co-existing congenital cardiac anomalies.

**EA type**	**Total number**	**Minor (no cardiac surgery)**	**Major (cardiac surgery)**
**A. Cohort (*****n*** **=** **84)**	**72/84 (86%)**	**57/72 (79%)**	**15/72 (21%)**
Type A	6/8 (75%)	6/6 (100%)	0/6 (0%)
Type B	5/8 (63%)	4/5 (80%)	1/5 (20%)
Type C	60/67 (90%)	46/60 (77%)	14/60 (23%)
Type D	1/1 (100%)	1/1 (100%)	0/1 (0%)
**B. Foker Process (*****n*** **=** **22)**	**19/22 (86%)**	**16/19 (84%)**	**3/19 (16%)**
Type A	5/7 (71%)	5/5 (100%)	0/5 (0%)
Type B	5/6 (83%)	4/5 (80%)	1/5 (20%)
Type C	9/9 (100%)	7/9 (78%)	2/9 (22%)
Type D	none	N/A	N/A

***Incidence of Congenital Cardiac Anomalies by Esophageal Atresia Types**. Table summarizes incidence of co-existing cardiac anomalies in infants born with esophageal atresia (EA) stratified by anatomical type in relation to the tracheo-esophageal fistula (See also Figure 1) for the entire cohort **(A)**, and in a subset of long-gap EA patients that underwent Foker process repair **(B)**. Incidence of cardiac anomalies is shown as total number (left column; n = 84). Of those that underwent cardiac surgery (n = 72), we summarize percent (%) of those with minor cardiac anomaly (not requiring surgery; central column; n = 57), and those that underwent cardiac repair (major anomaly; right column; n = 15)*.

### Severity Stratification of Underlying Disease

**Figure 3:**
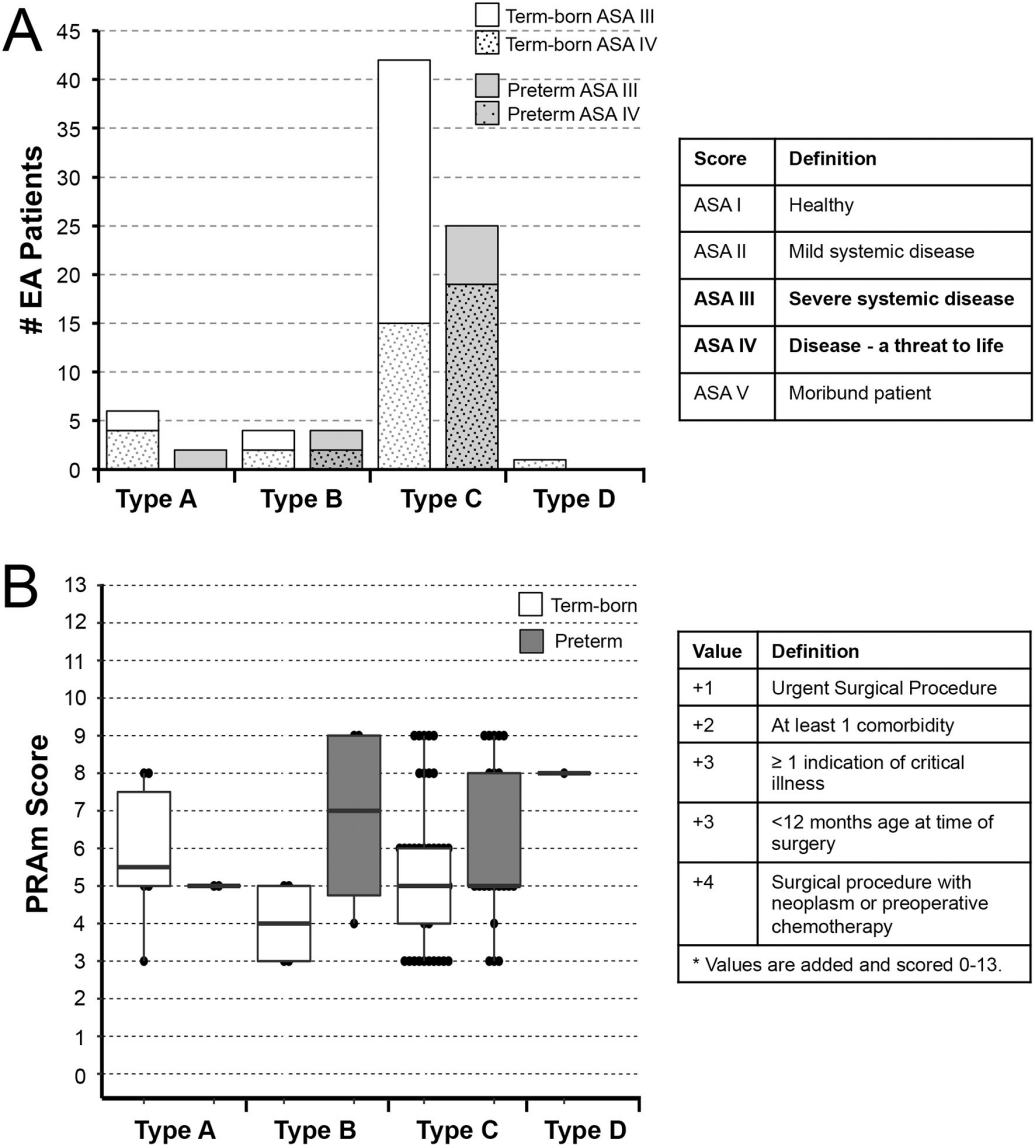
Underlying Disease Severity Stratification in Esophageal Atresia Cohort. Graphs illustrate incidence of American Society of Anesthesiologists (ASA) Physical Status classification **(A)** and Pediatric Risk Assessment (PRAm) severity scores **(B)** in infants born with esophageal atresia (EA) that underwent primary repair at a single institution from 2009-2020 (*n* = 84). Definition of scoring is shown in summary tables on the right for both the ASA Physical Status (16) and PRAm scoring (17–19). Graphs show stratification by anatomical types of EA (type A-D) and gestational age (term-born and premature; see Methods section). Specifically, all patients in this study were rated as either ASA Physical Status III or IV **(A)**. For the most common *type C* EA, the majority of term-born patients had ASA III status (27/42; 64%), while the majority of premature infants were assigned ASA IV status (19/25; 76%) implicating premature infants were more critically ill in the most common type of EA, *type C*. Graph in **(B)** illustrates distribution of PRAm scores per anatomical type and gestational age. Considering all infants had surgery when <12 months of age, the minimal score was 3. Since none of the infants had co-existing malignancy, the highest score was 9. From the graph in **(B)**, one can infer that premature infants had higher median score for type B EA, but lower median score for type C (thick horizontal line). Individual values are represented as dots, boxes span the interquartile range (IQR) (first and third quartile), and whiskers represent maximum and minimum values.

Figure 3 illustrates 2 different disease severity scores by anatomical EA types (types A-D; Figure 1B) and gestational age (term-born vs. premature).

#### American Society of Anesthesiologists Physical Status Classification

Half of the patients of this cohort were rated either ASA Physical Status III (49% (41/84) with severe systemic disease; 76% (31/41) term-born; 24% (10/41) premature), or ASA Physical Status IV (51% (43/84) with severe systemic disease that is a constant threat to life; 51% (22/43) term-born; 49% (21/43) premature). Figure 3A shows ASA Physical Status classification of the cohort infants per anatomical types of EA. For the most common type of EA – type C, the majority of term-born patients received ASA III status (27/42; 64%), while the majority of premature infants received ASA IV status (19/25; 76%).

#### Pediatric Risk Assessment Scores

**Figure 4:**
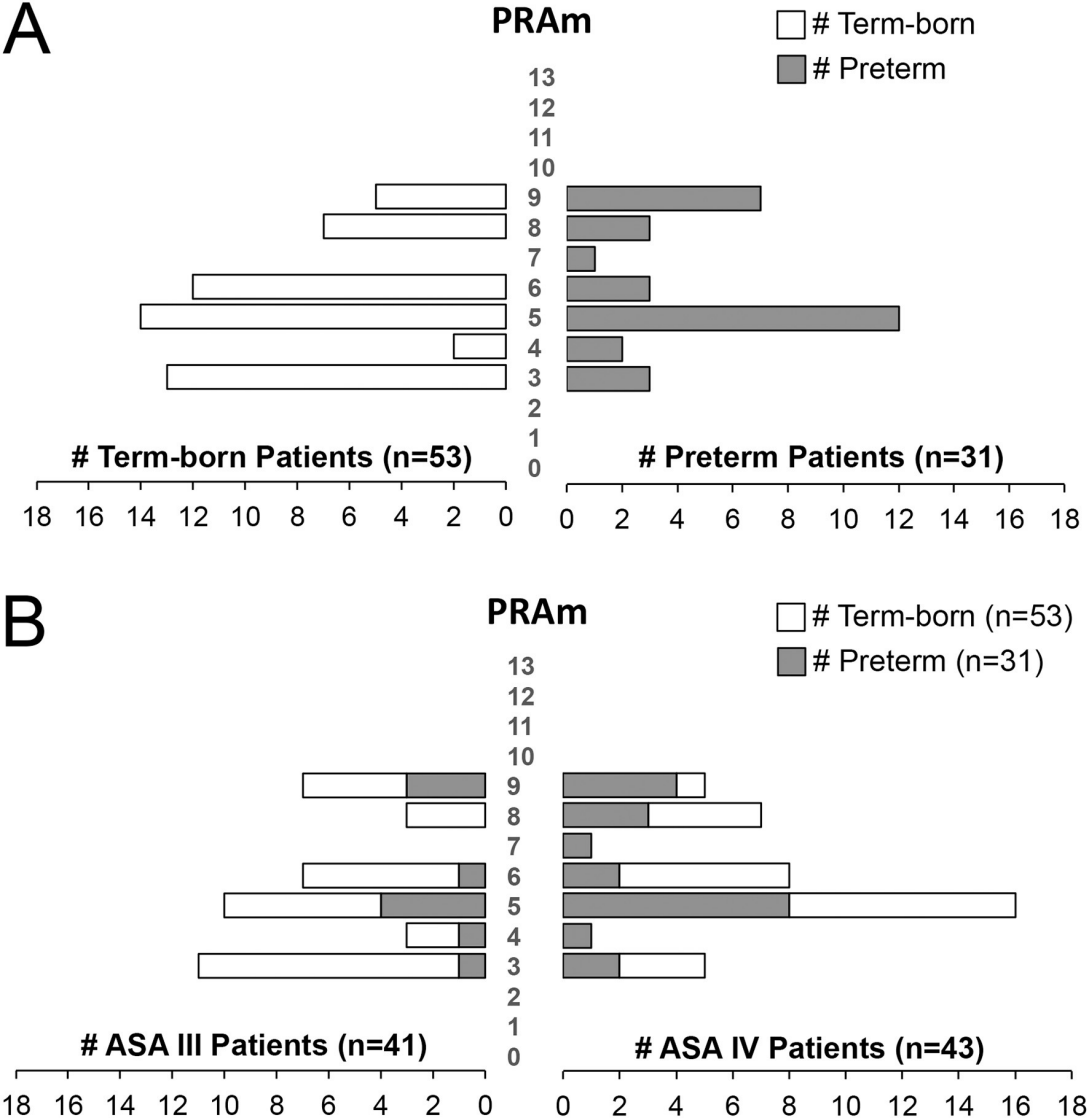
Pediatric Risk Assessment (PRAm) Scores in a Retrospective Cohort of Infants Born with Esophageal Atresia. **(A)** Shows PRAm scores of term-born (left) and preterm patients (right) illustrating a wide range of PRAm score across gestational age of infants born with esophageal atresia (EA; *n* = 84). Note a subtle tendency of term-born patients for lower, and premature patients for higher PRAm scores. **(B)** Plots PRAm scores in relation to American Society of Anesthesiologists (ASA) Physical Status Classification. Despite more premature infants having had higher ASA IV classification (21/31; 68%) in comparison to term-born (22/53; 41%; see also Figure 3A), PRAm score shows wide distribution of scores between 3 and 9. Such outlining is in support of ASA and not PRAm scoring in assessing disease severity when gestational age is the primary factor.

Considering all patients in this retrospective cohort underwent surgical repair in infancy, and none had any associated malignancy, the PRAm score ranged from 3 to 9 across all anatomical types and gestational age groups (Figure 3B). For the cohort as a whole, we report Median PRAm score of 5 for both term-born (interquartile range of 4–6) and premature infants (interquartile range of 5–8). When classified according to anatomical EA types, premature infants had a higher median score for type B EA (Median 7; interquartile range of 4.75–9), but a lower median score for most frequent type C (Median 5; interquartile range 5–8) in comparison to term-born infants (Median 4; interquartile range of 3–5 for type B; Median 5; interquartile range of 4–6 for type C). While type B shows higher PRAm scores for premature infants, there is a limited number of patients in this group (*n* = 8) compared to type C (*n* = 67). Furthermore, we report a wide PRAm score classification (Figure 4A) for term-born patients with propensity for lower PRAm scores (25% (13/53) PRAm 3; 9% (5/53) PRAm 9), and premature infants with propensity for higher PRAm scores (10% (3/31) PRAm 3; 23% (7/31) PRAm 9).

#### Relationship Between ASA Physical Status and PRAm Scores

To better gauge individual relationship of two different scores, Figure 4B illustrates relationship between ASA physical status and PRAm scores. Despite wide distribution of PRAm scores irrespective of the gestational age (Figure 4A), patients with assigned ASA IV classification had about equal distribution per gestational age groups: premature (21/43; 49%) and term-born patients (22/43; 51%). However, we do not show that premature infants with ASA IV physical classification (*n* = 21/43) align with higher PRAm score distribution (Figure 4B).

#### Disease Severity With Respect to Type of EA Surgical Repair

**Figure 5:**
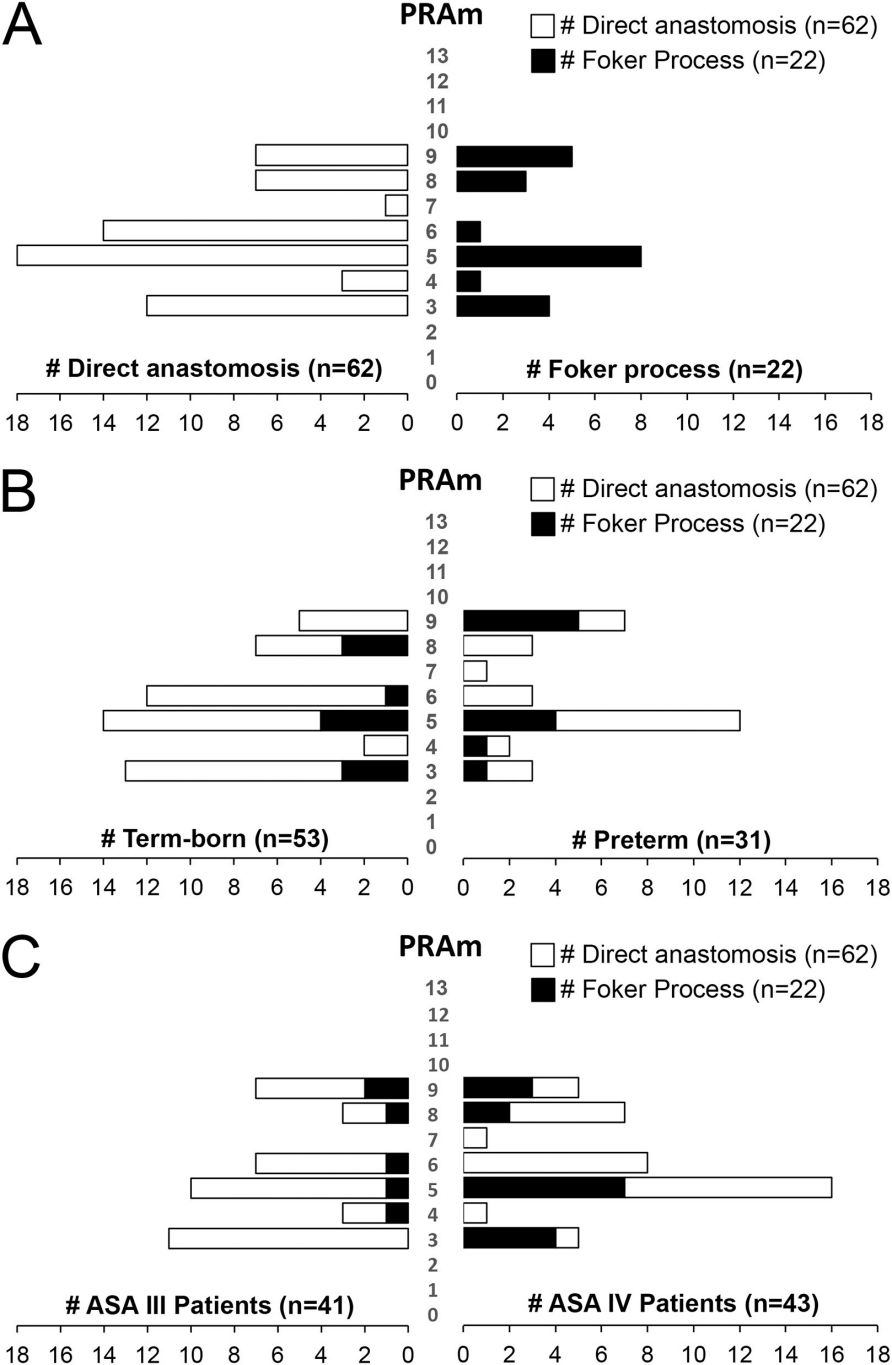
Disease Severity Stratification of Infants Born with Esophageal Atresia by Type of Surgical Repair. **(A)** Illustrates wide distribution of Pediatric Risk Assessment (PRAm) scores (from scores 3–9) by the type of surgical repair: (i) direct anastomosis for *short-gap* esophageal atresia (EA) repair (left), and (ii) Foker process (5–8) for *long-gap* EA repair (right). **(B)** Summarizes severity of disease per type of surgical repair in relation to the gestation age at birth. Similar to data in Figure 4A, note the subtle tendency of term-born patients having had lower, and premature patients higher PRAm scores. When PRAm scores are graphed in relation to American Society of Anesthesiologists (ASA) physical status **(C)**, we report more infants with *long-gap* EA with an ASA IV classification (73%; 16/22).

We also report a wide distribution of PRAm scores (scores 3-9) irrespective of the type of surgical repair (Figure 5A). However, infants born with *short-gap* EA undergoing direct anastomosis repair (*n* = 62) have a propensity for lower PRAm scores in term-born patients (62.5% (10/16) PRAm 3; 42% (5/12) PRAm 9) and higher PRAm scores in premature patients (12.5% (2/16) PRAm 3; 17% (2/12) PRAm 9) as illustrated in Figure 5B. Similarly, infants born with *long-gap* EA undergoing Foker process repair (*n* = 22) have a propensity for lower PRAm scores in term-born patients (19% (3/16) PRAm 3; 0% (0/12) PRAm 9) and higher PRAm scores in premature patients (6% (1/16) PRAm 3; 42% (5/12) PRAm 9) as illustrated in Figure 5B. Considering more premature patients with EA have ASA IV classification status (Figure 3A), prematurity should be considered a confounding factor for increased underlying disease severity. As such, other important aspects of prematurity, such as intra-uterine growth retardation and prematurity associated sequelae (e.g., respiratory distress syndrome) should be considered as potential indirect markers of prematurity in assessing outcomes following EA repair. Finally, when PRAm scores (with score range from 3–9) are graphed in relation to ASA physical status (Figure 5C), more infants with *long-gap* EA are scored as ASA IV classification (73%; 16/22) compared to *short-gap* EA patients (44%; 27/62). Future goals should include unique scoring system design that would include other potential confounders unique for EA repair, such as length of post-operative mechanical ventilation and antibiotic treatment as indirect markers of postoperative sedation and infections, respectively.

### Mortality Risk Assessment of Infants Born With Esophageal Atresia

#### Mortality Risk Assessment I

Table 3 summarizes the mortality risk assessment as originally described by Waterston et al. (21) in EA patients according to the (i) birth weight, (ii) co-existing congenital anomalies (Tables 1 and 2), and (iii) co-morbidity with pneumonia into: low (group A), moderate (group B), and high mortality risk (group C). Indeed, our retrospective study is of similar population size of EA patients (*n* = 84) as in the original report (*n* = 113) (21). However, our cohort had smaller numbers of infants in low risk group and much higher number of infants in high mortality group, group C (Table 4). With that in mind, we also show striking survival rates especially in the moderate risk (group B; 100% (42/42) vs. 68% (29/43 in the Waterson's study) and high risk group [group C; 95% (38/40) vs. 6% (2/32) in the original study (21)].

**Table 3:** Mortality risk assessment I.

**Group**	**Definition**	**1951–1959 (21) (*n* = 113)**	**2009–2020 (*n* = 84)**
A	Birth weight >2.5 kg	36/38 (95%)	2/2 (100%)
B	1.8 kg < Birth weight <2.5 kg or >2.5 kg with moderate congenital anomaly and/or pneumonia	29/43 (68%)	42/42 (100%)
C	Birth weight <1.8 kg or >2.5 kg with a severe congenital anomaly and/or pneumonia	2/32 (6%)	38/40 (95%)

***Survival rates of infants born with esophageal atresia according to scores by Waterson et al**. Using a modified Waterston et al. protocol (21), patients born with esophageal atresia (EA) were stratified into 3 categories **(A–C)** of mortality risk based on birth weight, co-existing anomalies, and presence or absence of pneumonia. The risk is described as low **(A)**, moderate **(B)**, and high **(C)** risk of mortality. Previously reported incidence of survival rates for EA patients (1951–1959) (21) is shown along with the current cohort's survival rates (2009–2020). Our results demonstrate increased survival, especially for group with moderate and higher mortality risk, groups B and C, respectively*.

**Table 4:** Mortality risk assessment II.

**Group**	**Definition**	**1980–1992 (20) (*n* = 372)**	**1993–2004 (27) (*n* = 188)**	**2009–2020 (*n* = 84)**
I	Birth weight >1.5 kg and no major cardiac anomaly	283/293 (97%)	130/132 (98.5%)	62/62 (100%)
II	Birth weight <1.5 kg or major cardiac anomaly	41/70 (59%)	41/50 (82%)	19/21 (90%)
III	Birth weight <1.5 kg and major cardiac anomaly	2/9 (22%)	3/6 (50%)	1/1 (100%)

***Survival Rates Assessment of Infants Born with Esophageal Atresia According to Scores by Spitz et al**. Using the Spitz et al. protocol (20), patients born with esophageal atresia (EA) were stratified into three categories of mortality risk based on birth weight, and the presence and severity of cardiac anomaly. Major cardiac anomalies were defined as one that required medical or surgical treatment. The risk is described as low **(I)**, moderate **(II)**, and high **(III)** risk of mortality. Spitz's original incidence of survival rates of EA patients for two separate decades, (1980–1992) (20) and (1993–2004) (27), are shown along with the current cohort's survival rates (2009–2020). Our results demonstrate increased survival, especially for low and moderate mortality risk groups*.

#### Mortality Risk Assessment II

The second scoring system by Spitz et al. (20) takes into account (i) birth weight and (ii) co-existence and (iii) severity of cardiac congenital anomalies. The risk is described as low (I), moderate (II), and high (III) risk of mortality. Although our retrospective cohort has lower power (*n* = 84) in comparison to previous decades' reports: 1980–1992 (*n* = 372) (20) and 1993–2004 (*n* = 188) (27), we report improved survival rates (Table 4), especially for low risk group (I; 100%; 62/62) and moderate risk group (II) of 90% (19/21). This data are in stark contrast to moderate risk group (II) survival rates of 59% (41/70) during 1980s (1980–1992) (20) and 82% (41/50) survival rate in the following decade (1993–2004) (27). Since our cohort only had only one patient that met criteria for group III - in part due to the exclusion criteria of extremely premature patients (<28 weeks), future clinical studies are needed to evaluate group III survival rates at our institution.

## Dicussion

Our novel results using retrospective approach from a single institution show near equal distribution of sex and gestational age (term-born vs. premature) by anatomical type of EA (types A–D) and by type of surgical repair (direct anastomosis vs. Foker process). We also share the incidence of co-occurring congenital anomalies with EA, with special emphasis on cardiac anomalies that have been shown to be a major mortality risk factor for infants born with EA (20). Although PRAm score showed a wider range of disease severity (3–9), ASA scores (III and IV) are more useful in predicting disease severity. We also report increased survival rate in our EA cohort in comparison to the literature in previous decades.

### Limitations of the Retrospective Chart Review

In keeping with the retrospective study design (28), data collected were originally intended for reasons other than research (29, 30). Due to the dependence of patient information stored for clinical practice, retrospective analysis may represent incomplete or missing documentation, poorly recorded or absent chart information, as well as difficult identification of desired patient data [e.g., attainment of PRAm scores (17) for cases of EA repair prior to 2017].

#### Study Size

Despite the exclusion of cases with extreme prematurity, and surgical repair at an outside institution, this study retained a moderate sample size with enough power to evaluate EA disease characteristics. The main challenges imposed with the exclusion criteria are that of generalizability since our institution represents a highly specialized level of care.

#### High-Risk Mortality Scores

Mortality risk assessment scores were quantified according to the scores previously described in the literature by Waterston et al. (21), and Spitz et al. (20). For both types of scoring, the high-risk groups used very low birth weights as surrogates for extreme prematurity. For this study, we defined Waterston risk assessment for the highest risk group (group C) as either (i) a low birth weight (associated with prematurity) or (ii) a standard birthweight with severe congenital anomaly or severe pneumonia. Therefore, analysis of Waterston et al. (21) group C was likely not significantly impacted by the exclusion of extreme prematurity. In contrast, the Spitz risk assessment required a birth weight <1.5 kg for the highest risk group (group III) for which only one patient from our retrospective cohort met criteria. Therefore, risk assessment of group III of the Spitz et al. (20) classification is not powered in our retrospective study.

### Characteristics of Esophageal Atresia Cohort

#### Anatomical Types of Esophageal Atresia

We report similar distribution of EA patients according to the anatomical classification (Figure 1A) compared to the literature (4, 31, 32) although one should keep in mind that definition of long-gap EA might differ. Our slightly increased incidence for type A and type B EA could be explained by the fact that data was obtained from a single institution, which pioneered the Foker process (5–8) for repair of *long-gap* EA (9) and receives patients locally, nationally and internationally. Indeed, we report higher incidence of *long-gap* EA in more rare types of EA: types A and type B, despite type C having the highest number of *long-gap* EA patients in this cohort (Figure 1D). The definitions for *long-gap* EA have not been agreed upon and can confound the comparison of data within the literature. Currently, a common method of classification is to simply define types A and B as *long-gap EA* and all others as *short-gap* EA (33, 34). The presence of long-gap EA patients with type C EA (Figure 1D) is in direct contrast to those studies that classify *long-gap* EA by purely anatomical classification. However, Ure et al. in 1995 (35) reported a total of 9 long-gap EA patients and a majority of them having type C EA. Study by Donoso et al. in 2016 (34) reported that a single patient with type C EA underwent *long-gap* EA repair despite being classified as *short-gap* in their study. Due to the inconsistency in the literature, we defined *long-gap* EA by surgical procedure [viz. Foker process (5–8)] implicating complex perioperative critical care instead of anatomical definitions based on the location of the EA gap.

#### Sex Distribution

We report nearly equal sex distribution for the entire cohort (*n* = 84), which is consistent with previous large retrospective reports (36–39). Since sex distribution for EA patients was only reported for the entire cohort (34, 39–41), we indicate – for the first time – that there is nearly equal sex distribution in EA patients by anatomical types (Figure 1C). We also report equal sex distribution for *long-gap* EA patients, which is in accordance with previous report in a larger cohort (9). Findings of equal sex distribution indicate that there is possibly no sex preference in infants born with EA – the subject of interest that continues to be investigated.

#### Distribution as Per Gestational Age

Our study found a slightly higher incidence of term-born patients in comparison to premature infants (28–37 weeks of gestation) with EA (Figures 2A,B). This finding stands in contrast to a large national cohort study of EA that included all gestational ages and found a higher prevalence of EA in premature patients (39). This discrepancy could be explained, in part, by our exclusion criteria that eliminated extremely premature patients from the cohort. However, our findings of higher incidence of term-born patients are consistent with the national studies in France (41) and Italy (40) reporting similar results. While the incidence of gestational age in *long-gap* EA patients are reported in the literature (6, 42), our novel results outline incidence of *long-gap* EA by anatomical types (Figure 1B). Discrepancy among gestational age within EA studies and novel findings of gestational age distribution in *long-gap* EA patients (with exclusion of extreme prematurity) suggests the need for future analysis to discern demographics of prematurity, as it represents an important risk factor in hospital mortality (43).

### Incidence of Associated Congenital Anomalies and Comorbidities With Esophageal Atresia

Congenital co-anomalies with EA can present as a wide spectrum across multiple organ systems (26), and can pose a challenge for care of infants with EA with increased risk of mortality and morbidity (44).

#### VACTERL Syndrome

The incidence of VACTERL association in this cohort was high at 37% compared to the literature report at around 10% (44) (Table 1A). Higher incidences of VACTERL syndrome in our report has previously been recognized in other studies (9) and is likely due to recognized differences in VACTERL diagnostics (45). Our cohort was comprised of sicker infants due to the very low incidence of isolated EA at 12% (Table 1B) compared to very large cross-hospital findings of isolated EA at 45% (36), 57.3% (37), and 38.7% (38) at other institutions. Findings of increased incidence of VACTERL and other comorbidities in this study possibly reflects institutional reputation as a national and international referral center for infants born with EA. Our report of higher incidence of VACTERL patients in *short-gap* EA are in accordance with previous report from our institution (9). This is in contrast to the study from Tabriz Children's Hospital and Tehran Mofid Hospital in Iran that reported no difference in incidence of VACTERL spectrum defects irrespective of the type of surgical repair required (42). The analysis of etiology of VACTERL syndrome is outside the scope of this study but is described well in the literature (25, 45, 46) and continues to be investigated.

#### Congenital Cardiac Anomalies

It is well known that co-occurring cardiac anomalies can impact the length and complexity of care for EA patients (41). We identified that most EA patients had co-existing cardiac anomalies: 86% for the entire cohort (Table 2A) and 79% in the non-syndromic cases (Table 1C). This is consistent with literature report from the Children's Hospital of Chongqing Medical University, China (71.2%) (47) and a large multicenter study of EA patients across 43 hospitals (70%) (43). We and others (48) report that a great majority of co-existing cardiac anomalies were defined as simple. We also report, for the first time, that only about a fifth of EA patients with co-existing cardiac anomalies had undergone cardiac repair – of which majority were type C EA cases (Table 2A). Our novel data in infants with *long-gap* EA show having nearly equal incidence of cardiac co-anomalies compared to the entire cohort with a similar incidence of patients requiring cardiac surgery (Table 2B). Our results confirm that (i) non-syndromic patients with EA may present with additional and potentially life-threatening congenital anomalies and that (ii) infants with non-syndromic *long-gap* EA can have cardiac anomalies imposing additional risk to their care (20, 41).

### Perioperative Risk Assessment

#### Underlying Disease Severity

Despite wider PRAm score variability (score 3-9; Figure 4A) and the same PRAm median score of 5 irrespective of the gestational age (Figure 3B), ASA Physical Status classification remains a golden standard in assessing underlying disease severity. We only noted a trend in term-born patients toward propensity for lower PRAm scores irrespective of the surgical type, while premature patients with *long-gap* EA showed propensity for higher PRAm scores (Figure 5B). Latter trends are in alignment with the seminal report in literature of a large cohort of infants undergoing non-cardiac surgery, validating PRAm scoring in predicting perioperative risk (17). Future work should also analyze unique risk factors related to surgical type of EA repair (viz. direct anastomosis vs. Foker process; open vs. laparoscopic approach) to expand on previous risk stratification of patients born with EA (49, 50). Morbidity risk assessment should also possibly include assessment of the neurological findings as our recent pilot study of infants with *long-gap* EA reported incidental brain findings for not only premature but term-born infants following Foker process repair (*n* = 13/group) (12, 15, 51, 52).

#### Mortality Risk Assessment

We report increased survival rates in this cohort as per two different mortality risk assessment scores. Despite our modification to the scoring schema by Waterston et al. (21), we report the total survival rates have vastly improved in the last decade for each of the described Waterston risk groups (Table 3). In addition, Spitz et al. (20) extended Waterston's mortality risk score in 1994 by including co-existing cardiac anomalies with EA. Indeed, the latter mortality risk score represents the most widely used mortality risk stratification that continues to be used for assessing risk in EA patients (27, 34). According to the most recent study of mortality predictors, major congenital heart disease was a significant predictor, while birth weight <1.5 kg was not (53). Indeed, we report increased survival rates in the most recent decade (2009–2020; Table 4) when compared to 1980s (20) and 1990s (27, 34) as previously published by Spitz et al. (20, 27). Such data are in support of great improvements in perioperative critical care of EA patients and treatment of their comorbidities, which may account for our reporting of improved survival. As per literature recommendation, the preferred clinical management of infants born with EA should be aided by highly specialized multidisciplinary team at expert centers (54) to help increase survival and decrease the incidence of morbidities (55). Therefore, improved outcomes in this report may be explained – in part, by the highly specialized nature of *The Esophageal and Airway Treatment Center* at our institution. Increased survival rates over the last decade suggest a potential need to assess unique operative and perioperative risks in this unique population of patients, as well as non-survival metrics such as functional status and quality of life.

Last, but not least, previous reports also suggests that extremely low birth weight infants with EA patients are at a higher risk of mortality (56), while a recent study showed potentially improved outcomes for extremely low birth-weight infants that underwent a staged repair for EA (57). Analysis of survival rates of extremely low birth weight infants with EA, especially those that underwent the Foker process for *long-gap* EA repair is needed to validate and potentially expand on these and our findings.

## Conclusions

We present a comprehensive analysis of EA patient classification by anatomical types (type A-D) and by surgical repair type (direct anastomosis vs. Foker process for *short-gap* vs. *long-gap* EA, respectively). Despite a wider PRAm score distribution in infants born with EA, ASA scores remain the gold standard in assessing underlying disease severity stratification. With increase in survival rates over the last decade, future studies should be directed toward assessing unique aspects of EA group in the context of (i) severity of underlying disease with and without comorbidities, (ii) unique complexities of perioperative critical care (with and without prolonged sedation, repeated procedures, and infection/sepsis assessment), and (iii) survival metrics such as functional status (e.g., neurobehavioral outcomes and quality of life). It is our hope that this retrospective study will be of service to research community when designing future clinical studies of risks and outcomes in this uniquely vulnerable population of infant patients.

## Data Availability Statement

The raw data supporting the conclusions of this article will be made available by the authors, without undue reservation.

## Ethics Statement

The Institutional Review Board at Boston Children's Hospital approved this retrospective cross-sectional research study (IRB-P000007855) of infants born with esophageal atresia (EA) that underwent primary surgical repair at a single institution.

## Author Contributions

Authorship credit was based on substantial contribution to (1) the conception and manuscript design (DE, JW, and DB), (2) acquisition (DE, JW, RJ, and DB), analysis (DE and DB), or interpretation of data (all authors), drafting the article (DE and DB) or critical revision for important intellectual content (all authors), (3) final approval of the version to be published (all authors), and (4) are accountable for all aspects of the work in ensuring that questions related to the accuracy or integrity of any part of the work are appropriately investigated and resolved (all authors).

## Funding

The Boston Children's Hospital 2019 OFD/BTREC/CTREC Faculty Career Development Fellowship, and 2017 Trailblazer Award from the Department of Anesthesiology, Critical Care and Pain Medicine, Boston Children's Hospital supported this work (DB). The content of this article is solely the responsibility of the authors and does not necessarily represent the official views of the funders.

## Conflict of Interest

The authors declare that the research was conducted in the absence of any commercial or financial relationships that could be construed as a potential conflict of interest.

## Publisher's Note

All claims expressed in this article are solely those of the authors and do not necessarily represent those of their affiliated organizations, or those of the publisher, the editors and the reviewers. Any product that may be evaluated in this article, or claim that may be made by its manufacturer, is not guaranteed or endorsed by the publisher.

## Abbreviations

ASA: American Society of Anesthesiologist
EA: Esophageal atresia
CHARGE: Coloboma, Heart defects, choanal Atresia, growth Retardation, Genital abnormalities, and Ear abnormalities
PRAm: Pediatric Risk Assessment
TEF: Tracheo-esophageal fistula
VACTERL: Vertebral, Anorectal, Cardiac, Tracheo-esophageal fistula and/or Esophageal atresia, Renal, and Limb defects/malformations.
